# Oxidatively damaged guanosine in white blood cells and in urine of welders: associations with exposure to welding fumes and body iron stores

**DOI:** 10.1007/s00204-014-1319-2

**Published:** 2014-08-09

**Authors:** Beate Pesch, Anne Lotz, Holger M. Koch, Boleslaw Marczynski, Swaantje Casjens, Heiko U. Käfferlein, Peter Welge, Martin Lehnert, Evelyn Heinze, Rainer Van Gelder, Jens-Uwe Hahn, Thomas Behrens, Monika Raulf, Andrea Hartwig, Tobias Weiss, Thomas Brüning

**Affiliations:** 1 Institute for Prevention and Occupational Medicine of the German Social Accident Insurance, Institute of the Ruhr-Universität Bochum (IPA), Bürkle-de-la-Camp-Platz 1, 44789 Bochum, Germany; 2 Institute for Occupational Safety and Health of the German Social Accident Insurance, IFA, Alte Heerstraße 111, 53757 Sankt Augustin, Germany; 3 Institute of Applied Biosciences, Karlsruhe Institute of Technology (KIT), Kaiserstraße 12, 76131 Karlsruhe, Germany

**Keywords:** Adducts, Chromium, Iron, Ferritin, Oxidative damage, Welders

## Abstract

The International Agency for Research on Cancer considers the carcinogenicity of welding fume of priority for re-evaluation. Genotoxic effects in experimental animals are still inconclusive. Here, we investigated the association of personal exposure to metals in respirable welding fumes during a working shift with oxidatively damaged guanosine in DNA of white blood cells (WBC) and in postshift urine samples from 238 welders. Medians of 8-oxo-7,8-dihydro-2′-deoxyguanosine (8-oxodGuo) were 2.35/10^6^ dGuo in DNA of WBC and 4.33 µg/g creatinine in urine. The median of 8-oxo-7,8-dihydroguanosine (8-oxoGuo) was 7.03 µg/g creatinine in urine. The extent of both urinary parameters was higher in welders applying techniques with high particle emission rates to stainless steel than in tungsten inert gas welders (8-oxodGuo: 9.96 vs. 4.49 µg/L, 8-oxoGuo: 15.7 vs. 7.7 µg/L), but this apparent difference diminished after creatinine adjustment. We applied random intercept models to estimate the influence of airborne and systemic exposure to metals on oxidatively damaged guanosine in WBC and urine together with covariates. We observed a highly significant nonlinear association of urinary 8-oxoGuo with serum ferritin (*P* < 0.0001) and higher 8-oxoGuo concentrations for respirable iron >1,000 µg/m^3^ compared to ≤57 µg/m^3^. Similar effects were found for manganese. Airborne chromium but not nickel was associated with all oxidatively modified guanosine measures, whereas urinary chromium as well as nickel showed associations with urinary modified guanosines. In summary, oxidatively damaged urinary guanosine was associated with airborne and systemic exposure to metals in welders and showed a strong relation to body iron stores.

**Electronic supplementary material:**

The online version of this article (doi:10.1007/s00204-014-1319-2) contains supplementary material, which is available to authorized users.

## Introduction

Welding is a commonly applied process to join metal parts, usually of mild or stainless steel. A large workforce is exposed to welding fumes, which mainly contain iron (Fe), but also manganese (Mn), chromium (Cr), and nickel (Ni) (Pesch et al. 2012; Weiss et al. 2013). In 1990, welding fumes were classified as “possibly carcinogenic” to humans (Group 2B) by the International Agency for Research on Cancer (IARC) (IARC Working Group on the Evaluation of Carcinogenic Risks to Humans 1990). Epidemiological evidence from pooled and meta-analyses has been accumulating that welders may show an excess lung cancer risk (Ambroise et al. 2006; Kendzia et al. 2013). An IARC expert group has recommended an update of the evaluation of the carcinogenicity of welding (Ward et al. 2010).

Fe and Mn are major constituents of welding fumes and tightly correlated with the overall particle concentration (Pesch et al. 2012). Cr and Ni are important additives to improve the quality of stainless steel; their concentrations in welding fumes are strongly correlated (Weiss et al. 2013). Hexavalent chromium (Cr(VI)) and Ni are classified as human carcinogens (IARC Working Group on the Evaluation of Carcinogenic Risks to Humans 1990). However, the observed excess lung cancer risk in welders could not be fully explained by welding of stainless steel and, hence, exposure to Cr(VI) or Ni only (Ambroise et al. 2006; Kendzia et al. 2013).

The inflammatory response to particulate matter in welding fumes may induce oxidative stress in the lung (Valavanidis et al. 2013). Besides the particle effect of welding fumes, the comprising metals, as for example Fe and Cr, may catalyze redox reactions, which can further increase the formation of reactive oxygen species (ROS) [reviewed in (Jomova and Valko 2011)]. Iron is the most abundant metal in the body, involved, for example, in oxygen transport and storage. Nevertheless, it is tightly regulated to avoid oxidative damage, with ferritin serving as iron storage protein (Wang et al. 2010). Serum ferritin (SF) is a recognized biomarker of body iron stores and has also been proposed to be linked to inflammation and cancer (Torti and Torti 2013). In a previous analysis, we found a weak association of SF with airborne Fe in welders (Pesch et al. 2012). Also, other metals present in welding fumes such as Ni, Cr, and Mn may be toxic and compromise genomic stability under overload conditions, partly due to the inactivation of defense systems against ROS and inhibition of DNA repair systems (Beyersmann and Hartwig 2008; Salnikow and Zhitkovich 2008).

Thus, metals can increase the extent of ROS either directly or indirectly, which can cause oxidative damage to DNA and other biomolecules. An oxidation product of guanosine, 8-oxo-7,8-dihydro-2′-deoxyguanosine (8-oxodGuo), is the most abundant and prominent DNA lesion. Oxidatively modified guanosine in the lung as target organ can be investigated in vivo in experimental animals, but not in healthy humans. Only noninvasive methods can be applied to collect body fluids as proxy tissue, for example, blood or urine (Valavanidis et al. 2009). The investigation of welders at their workplace requires highly robust methods to avoid accidental oxidative damage, particularly during the sampling, transport, storage, and processing of biological samples. Whereas 8-oxodGuo in white blood cells (WBC) is a critical biomarker due to stability and methodological challenges (Collins et al. 2004), urinary oxidatively damaged guanosines are rather stable and can be determined with robust and sensitive analytical methods (Marie-Desvergne et al. 2010; Ravanat 2012). The determination of 8-oxodGuo in WBC requires DNA isolation and is expressed as the fraction of oxidatively modified guanosine among all guanosines of the DNA strands. The determination of 8-oxodGuo in urine does not require a laborious DNA isolation and is expressed as mass concentration of oxidatively damaged nucleosides per L urine (Hu et al. 2014). Various sources of urinary 8-oxodGuo are possible, including mtDNA and free nucleoside pools in cells (Cooke et al. 2008). In addition to the assessment of oxidative damage to deoxyguanosines, also oxidatively modified guanosines from RNA turnover and metabolism can be determined in urine as 8-oxo-7,8-dihydro-2′-oxyguanosine (8-oxoGuo).

A quantitative exposure assessment has been addressed as pivotal in occupational epidemiology (Ward et al. 2010). Although millions of workers worldwide are exposed to welding fumes, only few and rather small studies measured respirable particles in the breathing zone of welders and explored their association with oxidatively damaged molecules together with pertinent covariates (Antonini 2003; Halasova et al. 2012). Here, we applied statistical modeling to estimate the impact of airborne and systemic exposure to metals on the occurrence of oxidatively modified guanosine in urine and blood samples from welders.

## Materials and methods

### Study population

The design of the WELDOX study has been previously described (Lehnert et al. 2012; Pesch et al. 2012; Weiss et al. 2013). Here, we analyzed 238 male welders of the WELDOX study recruited from 23 locations in Germany between 2007 and 2009. One welder with hematuria, one welder who donated blood within the last 2 days, and three welders with potentially interfering diseases were excluded from the original study population of 243 workers. For 21 other welders, no analyses of DNA adducts in WBC were available.

In short, usually twelve welders were recruited from each plant. During one working shift, four welders were equipped with personal air samplers on a Tuesday, Wednesday, or Thursday. Blood and urine were collected after shift, and information about the workplaces, smoking habits, and the medical history were documented in a questionnaire. All welders gave their written informed consent prior to examination. The study was conducted in accordance with the principles for medical research involving human subjects as defined by the Declaration of Helsinki. The local Ethics Committee of the Ruhr University Bochum approved the study protocol used in this study (Reg. No. 2732).

### Welding techniques and workplaces

Details about the welding process were documented according to a standardized format for monitoring German workplaces (Gabriel et al. 2010). The welding techniques at the day of examination included gas metal arc welding with solid wire (GMAW) (*N* = 96), flux-cored arc welding with shielding gas (FCAW) (*N* = 45), tungsten inert gas welding (TIG) (*N* = 65), and shielded metal arc welding (SMAW) (*N* = 19). Another 13 welders performed multiple techniques on the day of investigation. The physical workload of the welders was rated by the field-work team (Lehnert et al. 2012). Forty-seven welders had a high physical workload on the day of examination. Twenty-seven welders used a powered air-purifying respirator (PAPR), particularly when welding stainless steel with high-emission techniques. Although GMAW and FCAW were usually applied to mild steel, in one company, all welders used these techniques to weld stainless steel (Lehnert et al. 2014), subsequently referred to as “high-exposure group”.

### Sampling of respirable welding fumes and metal analysis

Welders were equipped with two personal samplers for collecting inhalable and respirable particles in the breathing zone inside of helmets as previously described (Lehnert et al. 2012). Here, we refer to the respirable particle fraction collected with a PGP-EA sampler at a flow rate of 3.5 L/min on cellulose nitrate filters (0.8 µm pore size, 37 mm diameter). The average duration of measurements was 3.5 h. The filters were shipped to the Institute for Occupational Safety and Health of the German Social Accident Insurance (IFA) for gravimetric determination of particulate matter and metal analysis by inductively coupled plasma mass spectrometry (ICP-MS) with a Perkin Elmer Elan DRC II (Waltham, Massachusetts) (Hebisch et al. 2005; Pesch et al. 2012; Weiss et al. 2013). The filters were digested with 10 mL of a mixture of nitric acid and hydrochloric acid. This solution was heated for 2 h under reflux. The mass spectrometer was calibrated with multielement standard solutions. Data from two samples were missing. The following numbers of measurements were below the limit of quantitation (LOQ): 88 (welding fume), 5 (Mn), 23 (Fe), 55 (Cr), and 76 (Ni). The mass of particulate matter collected on filters, and hence, the LOQs depend on airborne concentration in combination with sampling duration (Lehnert et al. 2012).

### Determination of metals in body fluids

Fe, Mn, Cr, and Ni were determined in blood and urine as described previously (Pesch et al. 2012; Weiss et al. 2013). The samples were shipped to the Institute for Prevention and Occupational Medicine of the German Social Accident Insurance (IPA) at 2–8 °C overnight. Urinary aliquots were stored at −20° C and EDTA blood aliquots at −80° C until processing. Samples were analyzed using graphite furnace atomic absorption spectrometry (ZEEnit 700; Analytik Jena, Jena, Germany). Interferences due to matrix effects were largely eliminated by means of ashing in the presence of oxygen, the Zeeman background compensation, and the standard addition procedure. Commercially available quality control material (RECIPE, Munich, Germany) was used to check the reliability of the analytical results. The LOQ was 1.0 µg/L for urinary Cr (CrU), 1.5 µg/L for urinary Ni (NiU), and 1.0 µg/L for Mn (MnB) and Fe (FeB) in blood. Within-series imprecision was better than 6.5 %, and between-days imprecision better than 10.5 %, respectively. A total of 106 measurements of CrU and 73 measurements of NiU were below LOQ. Accuracy of analytical results was ensured by successful participation in an international external quality assessment scheme for analyses in biological materials.

For the determination of Cr in erythrocytes, EDTA blood samples were stored at 2–8° C and processed within 24 h after blood collection. Erythrocytes were isolated from 5 mL whole blood by stepwise centrifugation and washing. The isolated erythrocytes were diluted with 2.5 mL ultrapure water, homogenized and cooled down to −20 °C for lysis and storage. About 250 µL of the erythrocyte solution was diluted with 250 µL 0.2 % Triton X in 0.1 % HNO_3_. The samples were analyzed using graphite furnace atomic absorption spectrometry (ZEEnit 700; Analytik Jena, Jena, Germany). Interference due to matrix effects was largely eliminated by means of ashing, the Zeeman background compensation, and the standard addition procedure. LOQ was 1.5 µg/L. Within-series imprecision was 3.0 %, and between-days imprecision 9.9 %. We detected Cr in erythrocytes of 15 blood samples; three samples could not be analyzed.

### Determination of 8-oxodGuo in white blood cells

EDTA blood samples were collected after shift, immediately frozen, and shipped on dry ice to the laboratory overnight. 8-OxodGuo was determined according to a published protocol (Marczynski et al. 1997, 2002). In brief, DNA was isolated from WBC at day of arrival and frozen at −80° C. The determination of 8-oxodGuo was performed with a Shimadzu HPLC/UV apparatus, connected to a Coulochem II (model 5200) electrochemical detector (ESA, Chelmosford, Mass., USA). The extent of oxidatively modified guanosine was measured as rate per 10^6^ 2′-deoxyguanoside (dGuo).

### Determination of 8-oxodGuo and 8-oxoGuo in urine

Urine samples were shipped to IPA overnight and stored at −80 °C until processing. Urinary 8-oxodGuo and 8-oxoGuo were determined with an online two-dimensional HPLC system coupled to a tandem mass spectrometer and quantified via isotope dilution. Analytical standards used for 8-oxodGuo were from Sigma–Aldrich, Germany, and for 8-oxoGuo from BIOZOL Diagnostica Vertrieb GmbH, Eching, Germany. Stable isotope-labeled standards used were [^15^N_5_]-8-oxodGuo from Cambridge Isotope Laboratories, Andover, USA, and [^13^C_5_]-8-oxoGuo, which was custom synthesized by Vladimir Belov, Max Planck Institute for Biophysical Chemistry, Göttingen, Germany.

In short, frozen urine samples were equilibrated to room temperature and homogenized by vortex mixing. A volume of 400 µL of each urine sample was spiked with 50 µL of the labeled internal standard solution (containing 200 µg/L labeled standard) and 200 µL ammonium acetate (1 M, pH 6,1). This mixture was frozen over night (to precipitate proteins), thawed, and centrifuged (10 min, 3,000 g). The supernatant was transferred into a new vial, and 100 µL was injected into the HPLC–MS/MS. Two-dimensional HPLC was carried out using a 1525 binary pump (loading pump) and a 1525µ binary pump (analytical pump) (Waters, Milford, USA), a Waters In-Line AF degasser and a Waters 2777 Sample Manager autosampler. In a two-column assembly, in principle described by Modick et al. (2013), a Capcell PAK 5u C18-MG-II (10 × 4.0 mm, 5 μm, Shiseido Co., Ltd, Japan) was used as first column for cleanup and enrichment. After automated backflush elution, chromatographic separation was performed on a Synergy Fusion-RP column (250 × 2.0 mm, 4 μm, Phenomenex, Germany). A water–methanol gradient (in 1 mM ammonium acetate pH 6.1 and 0.1 % formic acid) was used for elution with a flow rate of 0.25 mL/min. Cycle time between injections was 22 min.

Mass-spectrometric detection and quantification were performed using a Waters/Micromass Quattro Premier XE triple quadrupole mass spectrometer (Waters, Milford, USA) in the MRM mode with positive ionization. Mass transitions used for quantification were m/z 284 → 168 for 8-oxodGuo, m/z 300 → 168 for 8-oxoGuo, m/z 289 → 173 for [^15^N_5_]-8-oxodGuo, and m/z 305 → 168 for [^13^C_5_]-8-oxoGuo as previously described also by Marie-Desvergne et al. (2010). Linear calibration curves (between 0.25 and 50 µg nucleoside per liter urine) were obtained by plotting the quotients of peak area of the standards and the respective labeled internal standard as a function of concentration. LOQ for both nucleosides was 0.25 µg/L. Relative recoveries determined from eight different urines spiked with 2 and 8 μg/L of the nucleosides were between 82 and 118 %. Within-series and day-to-day imprecisions were below 10 %.

### Measurement of other variables

Smoking status was self-assessed and validated by urinary cotinine. Welders who reported to be a nonsmoker but showed urinary cotinine above 200 µg/L were categorized as current smokers. Urinary creatinine, SF, and other laboratory parameters were determined as formerly described (Henry et al. 2010; Pesch et al. 2012; Weiss et al. 2013). In brief, postshift samples of serum and K-EDTA whole blood were shipped to the laboratory overnight. SF, serum Fe, hemoglobin, and other variables were determined with standard methods on a Coulter LH 750 Analyzer (Beckman Coulter, Krefeld, Germany) at day of arrival.

### Statistical analysis

Medians and inter-quartile ranges (IQR) were used to describe the distributions of variables. For left-censored variables, an upper bound for the median and quartiles was presented. The Kendall rank correlation coefficient tau-b (*τ*_*b*_) was calculated as nonparametric measure of associations between left-censored variables (Isobe et al. 1986; Helsel 2005). To analyze potential predictors of oxidatively damaged guanosides, random intercept models (Snijders and Bosker 1999) were applied with welders in each plant as level-one units and plants as level-two units of variance estimates. The outcome of the model was the respective log-transformed adduct variable. Potential predictors were the internal and external exposure variables, age, and smoking status. Urinary damaged guanosides were additionally controlled for creatinine. The left-censored exposure variables were categorized. The fixed-effect estimators were re-transformed for presentation as factors that modify the level of oxidative damage. All calculations were performed with the statistical software SAS, version 9.3 (SAS Institute Inc., Cary, NC, USA).

## Results

### Oxidatively damaged guanosines in welders

Urinary 8-oxoGuo was determined with a median of 7.03 (IQR 5.99–9.01) µg/g creatinine in 238 welders. The median of urinary 8-oxodGuo was 4.33 (IQR 3.43–5.33) µg/g creatinine and strongly correlated with 8-oxoGuo (*τ*_*b*_ = 0.40, *P* < 0.0001). However, we could not detect an association between 8-oxodGuo in urine and WBC (*τ*_*b*_ = −0.07, *P* = 0.11). The characteristics of the welders and the distribution of adducts are presented in Supplementary Table 1. Correlations among adducts measures and exposure variables are shown in Supplementary Table 2.

**Fig. 1:**
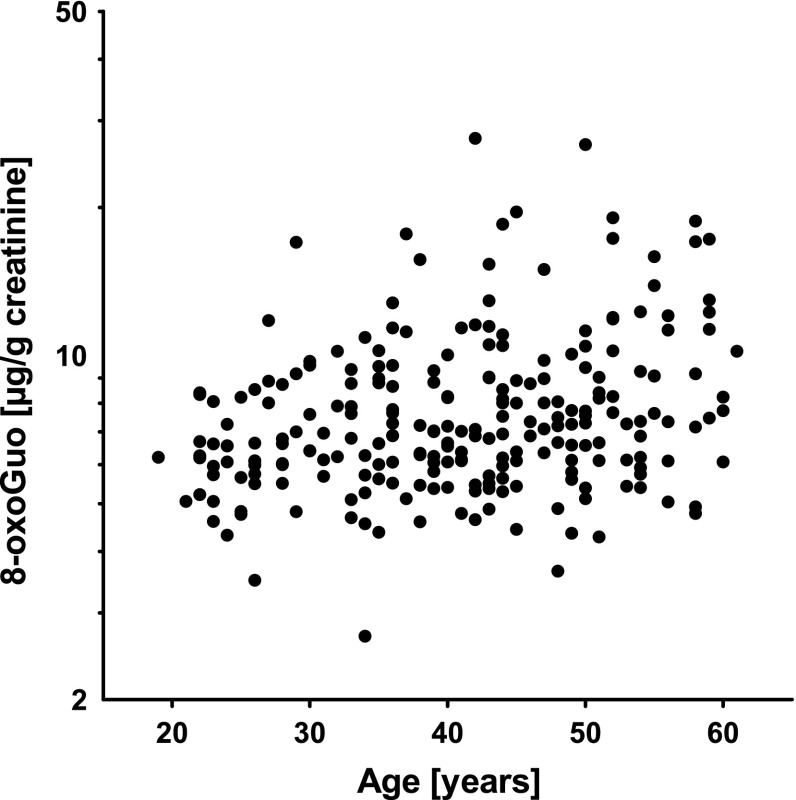
Association between age and urinary 8-oxo-7,8-dihydro-2′-oxyguanosine in 238 welders

**Fig. 2:**
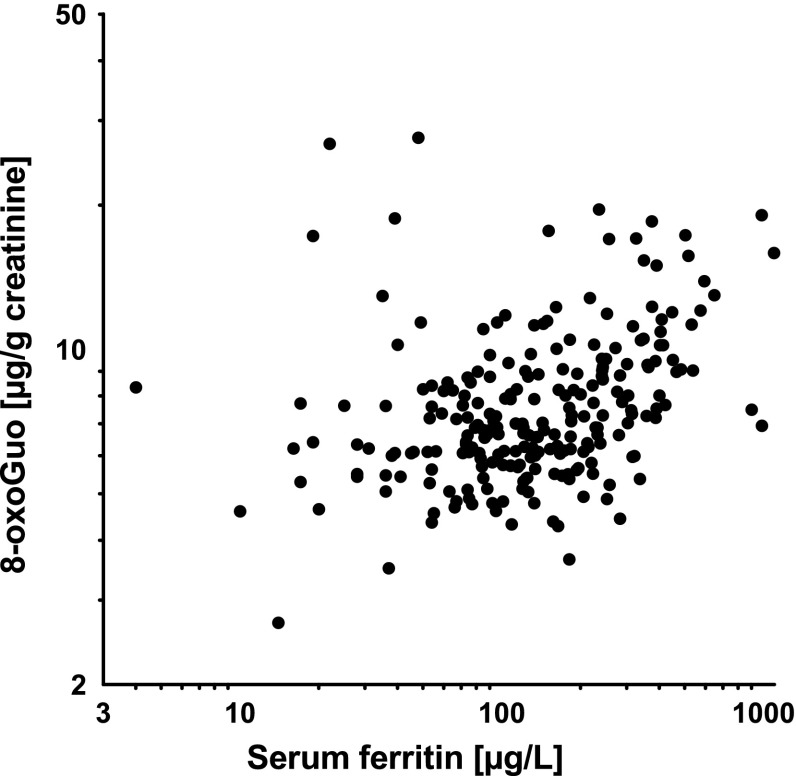
Association between serum ferritin and urinary 8-oxo-7,8-dihydro-2′-oxyguanosine in 238 welders

The median age of 238 welders was 42 years (range 19–61 years). More than 50 % of the welders reported active smoking. The univariate analysis of unadjusted data showed no clear association between oxidatively damaged guanosines and smoking status (Supplementary Table 1). Age and years of working as welder were tightly linked (*τ*_*b*_ = 0.68, *P* < 0.0001). Whereas we could not observe an accumulation of 8-oxodGuo by age, there was a trend of increasing excretion of 8-oxoGuo in urine by age as shown in Fig. 1. Figure 2 depicts the correlation of urinary 8-oxoGuo with body iron stores assessed as SF. Cr could be detected in erythrocytes of 15 (6.3 %) welders, who showed no obvious difference in the creatinine-adjusted concentrations of oxidatively modified guanosines compared to the other welders (e.g., 8-oxoGuo: 6.86 µg/g creatinine vs. 7.05 µg/g creatinine), although the unadjusted concentrations were higher (e.g., 8-oxoGuo: 16.44 vs. 10.99 µg/L). Few welders exhibited elevated C-reactive protein (*N* = 9) or reported an intake of acetylsalicylic acid medication (*N* = 10). We observed a minor increase in adduct levels in both subgroups of welders. Furthermore, we found lower DNA adduct rates in WBC in samples collected during winter.

### Oxidatively damaged guanosines by welding technique, material, and respiratory protection

Table 1 presents the distributions of adducts and exposure measures in all welders and for certain occupational exposure circumstances. The “high-exposure group” included welders who applied welding techniques with a high particle emission rate to stainless steel mostly in confined space. The median concentrations of respirable Cr (239 µg/m^3^) and urinary creatinine (2.00 g/L) ranked highest in this group compared to other settings. Eight out of these 12 welders had detectable Cr in erythrocytes. The second group comprised welders who applied FCAW to mild steel, where median exposure to airborne Fe and Mn as well as SF and Mn in blood were highest (Fe: 2,050 µg/m^3^, SF: 216 µg/L). By contrast, TIG welders and welders wearing a PAPR had frequently metal concentrations below LOQs. Median SF (103 µg/L) and urinary creatinine (1.13 g/L) were lowest in TIG welders. The distribution of oxidatively damaged guanosines in urine and WBC followed this ranking of settings with higher levels in welders applying high-emission techniques to stainless or mild steel than observed in TIG welders or when wearing a PAPR. However, this pronounced pattern diminished after creatinine adjustment of oxidatively modified urinary markers.

**Table 1:** Distribution of oxidative guanosine adducts and of exposure variables in all welders and in selected exposure circumstances

	Total			High-exposure group: gas metal arc welders with massive or flux-cored wire of stainless steel			Flux-cored arc welding of mild steel			Tungsten inert gas welders			Welders wearing a powered air-purifying respirator		
	*N* (*N* < LOQ)	Median	Interquartile range	*N* (*N* < LOQ)	Median	Interquartile range	*N* (*N* < LOQ)	Median	Interquartile range	*N* (*N* < LOQ)	Median	Interquartile range	*N* (*N* < LOQ)	Median	Interquartile range
Urinary 8-oxoGuo (µg/L)	238 (0)	11.1	(5.9; 16.1)	12 (0)	15.7	(11.0; 21.4)	30 (0)	13.8	(9.8; 17.2)	65 (0)	7.7	(3.9; 12.1)	27 (0)	11.5	(7.7; 15.1)
Urinary 8-oxoGuo (µg/g creatinine)	238 (0)	7.03	(5.99; 9.01)	12 (0)	6.59	(6.09; 8.39)	30 (0)	7.62	(5.97; 9.28)	65 (0)	6.41	(6.00; 8.25)	27 (0)	6.58	(5.45; 7.74)
Urinary 8-oxodGuo (µg/L)	238 (0)	6.19	(3.48; 9.58)	12 (0)	9.96	(6.08; 13.78)	30 (0)	6.02	(4.61; 8.06)	65 (0)	4.49	(2.46; 7.94)	27 (0)	6.85	(4.43; 9.38)
Urinary 8-oxodGuo (µg/g creatinine)	238 (0)	4.33	(3.43; 5.33)	12 (0)	4.28	(3.93; 5.34)	30 (0)	3.79	(2.84; 4.55)	65 (0)	4.41	(3.45; 5.15)	27 (0)	3.92	(3.23; 4.57)
8-oxodGuo/10^6^dGuo in white blood cells	217 (0)	2.35	(1.72; 3.94)	12 (0)	5.53	(4.44; 6.61)	30 (0)	4.79	(3.78; 6.77)	50 (0)	1.98	(1.67; 2.43)	27 (0)	1.92	(1.59; 3.36)
Respirable welding fumes (mg/m^3^)	236 (88)	0.94	(<0.42; 3.38)	12 (0)	4.99	(1.73; 8.04)	30 (0)	7.11	(4.53; 10.50)	65 (46)	<0.41	(<0.36; <0.50)	26 (25)	<0.40	(<0.36; <0.45)
Respirable manganese (µg/m^3^)	236 (5)	61	(<9; 300)	12 (0)	370	(191; 919)	30 (0)	775	(370; 1100)	65 (0)	9	(4; 22)	26 (5)	<4	(<0; 19)
Manganese in whole blood (µg/L)	238 (0)	10.3	(8.3; 13.2)	12 (0)	12.5	(11.2; 15.9)	30 (0)	13.5	(10.8; 16.0)	65 (0)	8.7	(7.1; 10.0)	27 (0)	10.7	(8.4; 12.8)
Respirable iron (µg/m^3^)	236 (23)	201	(31; 901)	12 (0)	513	(354; 858)	30 (0)	2,050	(1,100; 3,000)	65 (19)	22	(<11; 67)	26 (4)	<15	(<7; 31)
Serum iron (µg/dL)	238 (0)	91	(76; 107)	12 (0)	86	(69; 108)	30 (0)	92	(82; 107)	65 (0)	90	(72; 107)	27 (0)	94	(85; 109)
Serum ferritin (µg/L)	238 (0)	131	(77; 241)	12 (0)	120	(86; 207)	30 (0)	216	(121; 317)	65 (0)	103	(74; 164)	27 (0)	129	(85; 224)
Urinary iron (µg/L)	238 (10)	12.5	(8.7; 18.9)	12 (0)	12.4	(11.1; 15.4)	30 (0)	17.9	(13.2; 28.6)	65 (5)	9.4	(6.8; 14.2)	27 (0)	11.4	(8.5; 13.7)
Respirable chromium (µg/m^3^)	236 (55)	<3.8	(<1.8; 18.0)	12 (0)	239.0	(94.5; 373.0)	30 (11)	<1.2	(<0.7; <1.6)	65 (20)	<2.7	(<1.9; 6.8)	26 (6)	<3.5	(<2.0; <5.0)
Chromium in erythrocytes (µg/L)	235 (220)	<1.50	(<1.50; <1.50)	12 (4)	1.95	(<1.50; 2.37)	30 (30)	<1.50	(<1.50; <1.50)	65 (65)	<1.50	(<1.50; <1.50)	25 (25)	<1.50	(<1.50; <1.50)
Urinary chromium (µg/L)	238 (106)	1.19	(<1.00; 3.75)	12 (0)	13.53	(5.21; 53.03)	30 (21)	<1.00	(<1.00; 1.03)	65 (44)	<1.00	(<1.00; 1.21)	27 (9)	1.86	(<1.00; 4.42)
Respirable nickel (µg/m^3^)	236 (76)	<3.1	(<1.5; 16.5)	12 (0)	82.5	(52.5; 110.0)	30 (14)	<1.7	(<1.5; <3.1)	65 (25)	<1.5	(<1.0; <4.9)	26 (7)	<4.4	(<2.0; 7.2)
Urinary nickel (µg/L)	238 (73)	2.82	(<1.50; 6.01)	12 (0)	7.92	(5.78; 10.02)	30 (5)	2.70	(1.68; 4.08)	65 (38)	<1.50	(<1.50; 3.10)	27 (9)	3.55	(<1.50; 11.19)
Urinary creatinine (g/L)	238 (0)	1.53	(0.86; 2.10)	12 (0)	2.00	(1.55; 3.05)	30 (0)	1.68	(1.20; 2.13)	65 (0)	1.13	(0.58; 1.78)	27 (0)	1.80	(1.35; 2.15)

### Airborne and systemic exposure to metals as potential predictors of oxidatively damaged guanosine in welders

Welding fumes comprise two groups of tightly correlated constituents. One group consists of particulate matter, Fe, and Mn; the other group consists of Cr and Ni (Supplementary Table 2). We selected Fe as proxy for the first group and Cr as proxy for the second group to represent exposure to welding fumes in the statistical models together with smoking status and age as potential predictors of oxidatively damaged guanosine in WBC and urine (Table 2). We further substituted airborne by systemic exposure with SF and CrU (Table 3) and combined additionally Fe and SF with Ni and NiU, respectively (Tables 4, 5). The models with Mn and MnB in combination with Cr and CrU or Ni and NiU are shown as Supplementary Tables S3–S6. Urinary concentrations of oxidatively damaged guanosines were additionally controlled for creatinine due to the strong influence in all models (*P* < 0.0001). All models showed a variation in the levels of oxidatively damaged guanosines between the welders within plants and between plants.

**Table 2:** Influence of respirable iron and chromium and of other potential predictors on 8-oxodGuo and 8-oxoGuo in welders (random intercept models)

Fixed effects	Urinary 8-oxoGuo (µg/L) *N* = 236			Urinary 8-oxodGuo (µg/L) *N* = 236			8-oxodGuo/10^6^ dGuo *N* = 215		
	Exp (coefficient)	95 % CI	*P* value	Exp (coefficient)	95 % CI	*P* value	Exp (coefficient)	95 % CI	*P* value
Intercept	1.99	(1.04–3.81)	0.039	2.27	(1.14–4.51)	0.022	2.75	(1.29–5.84)	0.011
*Iron (µg/m* ^*3*^ *)*									
<LOQ (*N* = 23/23)	1.01	(0.82–1.24)	0.94	1.24	(0.99–1.56)	0.059	0.95	(0.73–1.23)	0.69
≥LOQ and ≤57 µg/m^3^ (*N* = 54/51)	1			1			1		
57–252 µg/m^3^ (*N* = 53/38)	1.04	(0.89–1.20)	0.64	1.04	(0.89–1.22)	0.62	1.01	(0.84–1.21)	0.93
252–1,055 µg/m^3^ (*N* = 53/50)	1.02	(0.88–1.18)	0.82	1.00	(0.85–1.18)	0.99	0.93	(0.77–1.13)	0.47
>1,055 µg/m^3^ (*N* = 53/53)	1.17	(1.01–1.35)	0.037	1.02	(0.87–1.20)	0.81	0.95	(0.77–1.17)	0.61
*Chromium (µg/m* ^*3*^ *)*									
<LOQ (*N* = 55/55)	0.85	(0.75–0.98)	0.022	0.83	(0.72–0.96)	0.012	0.83	(0.71–0.97)	0.021
≥LOQ and ≤6.8 µg/m^3^ (*N* = 91/83)	1			1			1		
>6.8 µg/m^3^ (*N* = 90/77)	1.00	(0.89–1.13)	0.99	0.98	(0.86–1.12)	0.77	1.04	(0.89–1.23)	0.61
Ln urinary creatinine (g/L)	2.56	(2.39–2.73)	<0.0001	2.48	(2.32–2.66)	<0.0001			
Active smokers (*N* = 121/110) versus nonsmokers (*N* = 115/105)	1.08	(0.99–1.18)	0.098	1.14	(1.03–1.25)	0.0081	0.96	(0.88–1.06)	0.43
Ln age (years)	1.42	(1.21–1.67)	<0.0001	1.19	(1.00–1.41)	0.056	1.00	(0.84–1.20)	0.97

Random effects	Variance component	95 % CI	*P* value	Variance component	95 % CI	*P* value	Variance component	95 % CI	*P* value
Level-two variance estimate (between plants)	0.010	(0.004–0.075)	0.072	0.021	(0.009–0.082)	0.028	0.25	(0.14–0.56)	0.0017
Level-one variance estimate (within plants)	0.104	(0.086–0.127)	<0.0001	0.112	(0.093–0.138)	<0.0001	0.11	(0.09–0.13)	<0.0001

**Table 3:** Influence of serum ferritin, urinary chromium, and other potential predictors on urinary 8-oxodGuo and 8-oxoGuo in welders (random intercept models)

Fixed effects	Urinary 8-oxoGuo (µg/L) *N* = 238			Urinary 8-oxodGuo (µg/L) *N* = 238			8-oxodGuo/10^6^ dGuo *N* = 217		
	Exp (coefficient)	95 % CI	*P* value	Exp (coefficient)	95 % CI	*P* value	Exp (coefficient)	95 % CI	*P* value
Intercept	4.30	(2.24–8.26)	0.0001	4.27	(2.10–8.68)	0.0003	2.66	(1.19–5.96)	0.020
Ln serum ferritin (mg/L)	1.14	(1.08–1.19)	<0.0001	1.11	(1.06–1.17)	<0.0001	1.00	(0.94–1.05)	0.87
*Urinary chromium (µg/L)*									
<LOQ (*N* = 106/103)	0.88	(0.77–1.00)	0.044	0.95	(0.83–1.09)	0.49	1.07	(0.91–1.25)	0.42
≥LOQ and ≤1.695 µg/L (*N* = 33/27)	1			1			1		
1.695–2.825 µg/L (*N* = 33/28)	1.00	(0.86–1.17)	0.99	1.08	(0.92–1.28)	0.34	1.09	(0.90–1.31)	0.38
2.825–7.850 µg/L (*N* = 33/31)	1.19	(1.01–1.39)	0.039	1.21	(1.01–1.44)	0.037	1.13	(0.92–1.39)	0.25
>7.850 µg/L (*N* = 33/28)	1.12	(0.95–1.32)	0.16	1.14	(0.95–1.36)	0.16	1.15	(0.93–1.43)	0.20
Ln urinary creatinine (g/L)	2.37	(2.21–2.53)	<0.0001	2.32	(2.16–2.50)	<0.0001			
Current smokers (*N* = 122/111) versus nonsmokers (*N* = 116/106)	1.07	(0.98–1.16)	0.13	1.14	(1.04–1.24)	0.0058	0.97	(0.88–1.07)	0.55
Ln age (years)	1.26	(1.08–1.47)	0.004	1.05	(0.89–1.24)	0.58	0.97	(0.81–1.17)	0.74

Random effects	Variance component	95 % CI	*P* value	Variance component	95 % CI	*P* value	Variance component	95 % CI	*P* value
Level-two variance estimate (between plants)	0.009	(0.004–0.051)	0.048	0.013	(0.005–0.061)	0.039	0.24	(0.14–0.50)	0.0011
Level-one variance estimate (within plants)	0.092	(0.076–0.112)	<0.0001	0.108	(0.090–0.132)	<0.0001	0.11	(0.09–0.14)	<0.0001

**Table 4:** Influence of respirable iron and nickel and of other potential predictors on 8-oxodGuo and 8-oxoGuo in welders (random intercept models)

Fixed effects	Urinary 8-oxoGuo (µg/L) *N* = 236			Urinary 8-oxodGuo (µg/L) *N* = 236			8-oxodGuo/10^6^ dGuo *N* = 215		
	Exp (coefficient)	95 % CI	*P* value	Exp (coefficient)	95 % CI	*P* value	Exp (coefficient)	95 % CI	*P* value
Intercept	1.97	(1.02–3.78)	0.044	2.29	(1.14–4.58)	0.022	2.73	(1.28–5.82)	0.012
*Iron (µg/m* ^*3*^ *)*									
<LOQ (*N* = 23/23)	0.93	(0.76–1.13)	0.45	1.15	(0.93–1.44)	0.20	0.89	(0.69–1.16)	0.40
≥LOQ and ≤57 µg/m^3^ (*N* = 54/51)	1			1			1		
57–252 µg/m^3^ (*N* = 53/38)	1.00	(0.87–1.16)	0.98	1.01	(0.87–1.18)	0.89	1.00	(0.84–1.19)	1.00
252–1,055 µg/m^3^ (*N* = 53/50)	0.96	(0.82–1.12)	0.56	0.95	(0.80–1.12)	0.54	0.91	(0.75–1.10)	0.32
>1,055 µg/m^3^ (*N* = 53/53)	1.14	(0.98–1.32)	0.085	1.01	(0.85–1.19)	0.95	0.97	(0.80–1.19)	0.78
Nickel (µg/m^3^)									
<LOQ (*N* = 76/75)	0.97	(0.86–1.09)	0.59	0.92	(0.81–1.06)	0.25	0.93	(0.80–1.09)	0.39
≥LOQ and ≤8.4 µg/m^3^ (*N* = 81/67)	1			1			1		
>8.4 µg/m^3^ (*N* = 79/73)	1.08	(0.95–1.22)	0.24	1.04	(0.90–1.19)	0.61	1.08	(0.92–1.27)	0.33
Ln urinary creatinine (g/L)	2.57	(2.41–2.75)	<0.0001	2.50	(2.33–2.68)	<0.0001			
Active smokers (*N* = 121/110) versus nonsmokers (*N* = 115/105)	1.09	(0.99–1.19)	0.072	1.14	(1.04–1.26)	0.0063	0.97	(0.88–1.07)	0.59
Ln age (years)	1.42	(1.20–1.67)	<0.0001	1.18	(0.99–1.40)	0.068	1.00	(0.83–1.20)	0.97

Random effects	Variance component	95 % CI	*P* value	Variance component	95 % CI	*P* value	Variance component	95 % CI	*P* value
Level-two variance estimate (between plants)	0.010	(0.003–0.075)	0.076	0.021	(0.009–0.082)	0.027	0.24	(0.14–0.52)	0.0015
Level-one variance estimate (within plants)	0.106	(0.088–0.130)	<0.0001	0.115	(0.095–0.141)	<0.0001	0.11	(0.09–0.14)	<0.0001

**Table 5:** Influence of serum ferritin, urinary nickel, and other potential predictors on urinary 8-oxodGuo and 8-oxoGuo in welders (random intercept models)

Fixed effects	Urinary 8-oxoGuo (µg/L) *N* = 238			Urinary 8-oxodGuo (µg/L) *N* = 238			8-oxodGuo/10^6^ dGuo *N* = 217		
	Exp (coefficient)	95 % CI	*P* value	Exp (coefficient)	95 % CI	*P* value	Exp (coefficient)	95 % CI	*P* value
Intercept	3.56	(1.85–6.88)	0.0006	3.84	(1.90–7.77)	0.0007	2.81	(1.27–6.22)	0.014
Ln serum ferritin (mg/L)	1.14	(1.08–1.19)	<0.0001	1.11	(1.06–1.17)	<0.0001	1.00	(0.94–1.05)	0.85
*Urinary nickel (µg/L)*									
<LOQ (*N* = 73/68)	0.94	(0.83–1.06)	0.31	1.02	(0.89–1.17)	0.82	0.93	(0.80–1.09)	0.36
≥LOQ and ≤2.63 µg/L (*N* = 42/40)	1			1			1		
2.63–4.12 µg/L (*N* = 41/38)	1.04	(0.90–1.20)	0.57	0.95	(0.82–1.11)	0.54	1.12	(0.95–1.31)	0.18
4.12–8.04 µg/L (*N* = 41/37)	1.15	(1.00–1.33)	0.052	1.10	(0.94–1.28)	0.25	0.96	(0.81–1.14)	0.65
>8.04 µg/L (*N* = 41/34)	1.23	(1.05–1.44)	0.012	1.24	(1.03–1.49)	0.021	0.95	(0.76–1.18)	0.61
Ln urinary creatinine (g/L)	2.37	(2.21–2.54)	<0.0001	2.36	(2.19–2.54)	<0.0001			
Current smokers (*N* = 122/111) versus nonsmokers (*N* = 116/106)	1.06	(0.98–1.16)	0.16	1.14	(1.04–1.25)	0.0039	0.96	(0.87–1.05)	0.36
Ln age (years)	1.30	(1.11–1.52)	0.0013	1.07	(0.91–1.27)	0.42	0.98	(0.82–1.18)	0.86

Random Effects	Variance component	95 % CI	*P* value	Variance component	95 % CI	*P* value	Variance component	95 % CI	*P* value
Level-two variance estimate (between plants)	0.007	(0.003–0.062)	0.082	0.019	(0.009–0.062)	0.015	0.23	(0.13–0.48)	0.0011
Level-one variance estimate (within plants)	0.095	(0.079–0.116)	<0.0001	0.105	(0.087–0.128)	<0.0001	0.11	(0.09–0.13)	<0.0001

Except for respirable Ni (Table 4), we observed nonlinear associations of urinary 8-oxoGuo with airborne Fe, Cr, and Mn (Tables 2, S3). For example, welders exposed to Fe > 1,055 µg/m^3^ or Mn > 320 µg/m^3^, respectively, showed higher 8-oxoGuo concentrations than welders exposed to Fe ≤ 57 µg/m^3^ (*P* = 0.04) or Mn ≤ 9.7 µg/m^3^, respectively (*P* = 0.03). Regarding systemic exposure, we observed a significant nonlinear association of urinary concentrations of oxidatively damaged guanosines with SF (*P* < 0.0001) (Table 3; Fig. 2) and a weaker relation between MnB and 8-oxoGuo (*P* < 0.05) (Table S4 and S6). We further found associations of urinary oxidatively damaged guanosine with CrU (Table 3) and NiU (Table 5). The multivariate models confirmed age as predictor of urinary 8-oxoGuo (see also Fig. 1) and revealed that active smokers had higher concentrations of urinary 8-oxodGuo than nonsmokers (e.g., Table 5).

## Discussion

Several millions of workers worldwide are exposed to welding fumes. A unifying process in particle and metal toxicology is the generation of ROS and oxidatively derived damage of biological molecules (Valko et al. 2006). Particulate matter, Fe, Cr, and other metals in inhaled welding fumes may induce the formation of ROS and thus oxidize nucleobases such as guanine of RNA and DNA or from the intracellular nucleotide pool. In the WELDOX study, we determined oxidatively damaged guanosines as 8-oxodGuo in relation to the number of unmodified guanosines in isolated nuclear DNA from WBC and in urine as the mass concentration per mL urine as 8-oxodGuo and 8-oxoGuo from various sources of DNA and RNA metabolism. We applied statistical modeling to determine airborne and internal exposure to metals in welding fumes as potential predictors of the levels of oxidatively damaged guanosines together with pertinent covariates in 238 welders. We observed a highly significant association of urinary 8-oxodGuo and 8-oxoGuo with SF in welders. The correlation of body iron stores with urinary 8-oxodGuo in welders is in line with a report on a strong and robust association in the general population (Hori et al. 2010). Furthermore, we detected weak and nonlinear associations of respirable Cr with all measures of oxidatively modified guanosine as biomarkers of oxidative stress and of all systemic metal concentrations with urinary 8-oxoGuo.

Welding fumes are complex mixtures, which contain particulate matter with Fe and Mn as the most abundant metals when joining parts of steel. Welding of mild steel is commonly associated with high concentrations of particles, Fe and Mn (Lehnert et al. 2012; Pesch et al. 2012; Weiss et al. 2013). By contrast, welding of stainless steel is related to lower concentrations of particles, Fe and Mn. Cr and Ni are additionally emitted from consumable electrodes if they contain these metals, and when welding stainless steel. The tight statistical correlations between airborne Fe and Mn (Pesch et al. 2012) on the one hand and between Cr and Ni (Weiss et al. 2013) on the other hand do not allow a “causal” attribution of genotoxic effects to a certain airborne metal selected for statistical modeling. The strong relations between certain welding fume constituents do not hold for measures of internal exposure to these metals. Overall, we found no linear association between SF and MnB and only a weak correlation between CrU and NiU (Pesch et al. 2012; Weiss et al. 2013), which may be due to a tightly controlled homeostasis of essential trace elements. In addition, more subtile and complex associations may occur, such as the competition for transporter systems as has been shown in case of Fe and Mn (Gunshin et al. 1997). We did not find an indication of an association of airborne Ni with oxidative damage but a weak association with urinary Ni, which may reflect residual correlation with CrU. Nickel has a lower capacity of ROS induction than Cr(VI), but may deplete antioxidants and induce hypoxia [for review (Cameron et al. 2011)]. Notably, oxidatively modified nucleobases do not represent the full spectrum of possible genotoxic effects of welding fume.

Strengths of our cross-sectional study are the comprehensive data collected in a large group of welders, personal measurements of respirable welding fume in the breathing zone together with measures of systemic exposure, and statistical modeling to estimate exposure effects adjusted for potential confounders. We observed slightly higher urinary 8-oxoGuo concentrations in welders exposed to respirable Fe above about 1 mg/m^3^ in addition to a highly significant association of body iron stores with urinary concentrations of both 8-oxoGuo and 8-oxodGuo. SF as a recognized biomarker of body iron stores was associated with airborne iron in a nonlinear manner, indicating homeostasis until a certain level of inhalative iron exposure (Pesch et al. 2012). These observations are suggestive for a role of iron overload in oxidatively derived damage to guanosine from DNA and RNA metabolism in a subgroup of welders exposed to high iron concentrations. So far, prospective lung cancer studies in welders with biomarkers of iron metabolism and oxidative stress are still lacking to assess their cancer-predictive value (Fonseca-Nunes et al. 2014).

The highly significant association between SF and urinary concentrations of oxidatively modified guanosine cannot fully be disentangled regarding the role of particles and iron in welding fumes. SF is also a biomarker for inflammation [for review (Wang et al. 2010)] and may include an inflammatory response to particle exposure in welders. Evidence for a role of iron in carcinogenesis is mounting, and cancer has been considered a ferrotoxic disease (Toyokuni 2009). Iron is an essential nutrient but also a redox-active metal where a disturbed homeostasis has been associated with oxidatively derived damage, inflammation, and cancer (Torti and Torti 2013). Even though epithelial cells in lungs are equipped with ferritin (Ghio et al. 2006), very high airborne exposure to respirable Fe may overload the tightly regulated iron homeostasis (Pesch et al. 2012). A detailed exploration of the iron status of welders is beyond the scope of this article but subject of another analysis.

Epidemiological studies did not reveal a clear difference in lung cancer risk of welders using stainless compared to mild steel, and hence, exposure to Cr(VI) or Ni does not sufficiently explain the observed excess risks (Ambroise et al. 2006; Kendzia et al. 2013). In this cross-sectional study among welders, the effect of respirable and urinary Cr on oxidatively damaged guanosine in urine and WBC was rather weak. Airborne Cr is mainly trivalent in most welding settings and cannot easily enter (blood) cells. A major limitation was that we could not determine hexavalent Cr, because the welders were already equipped with two devices for sampling inhalable and respirable particles in parallel (Lehnert et al. 2012). The analytical determination of Cr(VI) is challenging, especially in welding fumes due to other interfering metal oxides (Unceta et al. 2010). Inhaled Cr(VI) may be further reduced in the breathing zone (De Flora 2000) and respiratory tract (Izzotti et al. 1998). We used the detection of Cr in erythrocytes to assess exposure to Cr(VI), which can enter cells through anion channels [for review (Nickens et al. 2010)]. However, Cr could only be detected in erythrocytes of 6 % of all welders. This group comprised several welders applying SMAW, which are known to be potentially exposed to Cr(VI) (Emmerling et al. 1990). Only in the “high-exposure group”, the majority of welders had detectable Cr in erythrocytes. They applied GMAW or FCAW to stainless steel mostly in confined space (Lehnert et al. 2014). Their concentrations of oxidatively damaged guanosine were higher than in TIG welders or when wearing a PAPR for respiratory protection. In this particular high-exposure setting, also urinary creatinine was higher than in TIG welders, indicating heavy physical work.

The “oxidative status” of a welder is an individual balance between the generation of ROS by normal cellular metabolism, exposure to welding fume and lifestyle factors, and the capacity of antioxidant and defense mechanisms. A general advantage of the determination of oxidatively modified guanosines in urine is that the methods are considered less susceptible to artifactual oxidation than the determination of DNA adducts in WBC (Cooke et al. 2008; Il’yasova et al. 2012). Furthermore, (tandem) mass-spectrometric methods with quantification via isotope dilution are both highly selective and sensitive (Barregard et al. 2013). However, a large fraction of the variance of oxidative damage remained unexplained in all models. So far, less is known about the sources of urinary 8-oxoGuo (Cooke et al. 2008). This raises the questions whether the excretion of degradation products into urine may also reflect at least partially clearance mechanisms in addition to exposure. A controversial issue in human biomonitoring is creatinine adjustment (Pesch et al. 2011; Aylward et al. 2014), which is a classical approach to correct for urine dilution. However, urinary creatinine may depend upon physical activity, but also on age, sweating, drinking habits, and other factors (Umweltbundesamt 1998). Welders with a high physical workload excreted higher concentrations of creatinine and had considerably higher adduct concentrations than welders with a low physical workload. This raises the question whether physical activity may contribute to both, elevated concentrations of urinary creatinine and oxidatively modified guanosine. The higher concentrations of oxidatively damaged guanosine in welders with Cr in erythrocytes compared to welders without Cr in erythrocytes diminished to some degree when urinary adducts were adjusted for creatinine in a simple manner as ratio of both concentrations. Our multivariate models included in addition to creatinine also age and smoking in combination with measures of exposures, because the excretion of 8-oxodGuo into urine was slightly higher in active smokers and 8-oxoGuo increased by age. When adjusting for these covariates, the estimate’s effects are supportive for an influence of welding fume on urinary 8-oxoGuo and 8-oxodGuo. This influence is further supported by the strong association of SF with oxidatively modified guanosine in urine, although an attribution of the effects to particulate matter and the various metals in welding fumes remains to be elucidated.

We further assessed whether high levels of oxidative damage may have been caused by factors not captured in the model. The highest DNA adduct rate in WBC was observed in a welder who donated blood 2 days prior to examination. Subjects with blood donation prior to blood drawing were excluded from this analysis. Slightly elevated adduct rates in WBC were determined in few welders using acetylsalicylic acid as medication. The latter observation is in line with in vitro studies where acetylsalicylic acid induced apoptosis by increasing the production of ROS (Farrugia et al. 2013; Raza et al. 2011).

A lack of a clear association of exposure with 8-oxodGuo in WBC has been observed in this and former studies (Pesch et al. 2007). Excellent reviews claim problems with artifactual oxidation and large differences between methods when determining 8-oxoGuo in WBC (Collins et al. 2004; Valavanidis et al. 2009). Besides analytical challenges when analyzing 8-oxodGuo in DNA isolated from WBC, it has to be taken into account that WBC comprise a mixture of different cell types, with different life-spans, which employ different strategies for the uptake, storage, and release of iron [for review (Weiss 2005)]. For example, lymphocytes have a low ferritin content and seem to support the body’s retention of iron for pathogens (Wang et al. 2013). By contrast, macrophages and monocytes capture particulate matter, recycle iron from senescent erythrocytes, and have a high ferritin content. This complexity may require a separate analysis of the different WBC cell populations in terms of measuring oxidative damage.

In conclusion, high exposure to respirable iron and the concentrations of SF as biomarker of body iron stores were associated with the excretion of 8-oxodGuo und 8-oxoGuo into the urine of welders. We further found higher levels of 8-oxoGuo in welders with detectable concentrations of Cr in welding fumes as well as in erythrocytes. Welders are exposed to a complex mixture of particles and redox-active metals where efficient ventilation and respiratory protection should be provided as preventive measure to reduce the exposure and oxidative stress.

## Electronic supplementary material

Below is the link to the electronic supplementary material.

Supplementary material 1 (DOCX 25 kb)

Supplementary material 2 (DOCX 24 kb)

Supplementary material 3 (DOCX 20 kb)

Supplementary material 4 (DOCX 20 kb)

Supplementary material 5 (DOCX 20 kb)

Supplementary material 6 (DOCX 20 kb)
