# Supplementary material for: Oxidatively damaged guanosine in white blood cells and in urine of welders: associations with exposure to welding fumes and body iron stores
Source: Arch Toxicol. 2014 Aug 9;89(8):1257–69. doi: 10.1007/s00204-014-1319-2 (PMC4508371; doi:10.1007/s00204-014-1319-2)
Supplement: Supplementary file 1 — Supplementary material 1 (DOCX 25 kb) [file 204_2014_1319_MOESM1_ESM.docx]

**Table S1: Distribution of guanosine adducts in urine and white blood cells by characteristics of the welders**

| Characteristics | | Urine | | | | | | White blood cells | | |
| --- | --- | --- | --- | --- | --- | --- | --- | --- | --- | --- |
|  |  | 8-oxoGuo µg/g creatinine | | | 8-oxodGuo µg/g creatinine | | | 8-oxodGuo/10^6^dGuo | | |
|  |  | N | Median | Interquartile range | N | Median | Interquartile range | N | Median | Interquartile range |
| Total |  | 238 | 7.03 | (5.99; 9.01) | 238 | 4.33 | (3.43; 5.33) | 217 | 2.35 | (1.72; 3.94) |
|  | | | | | | | | | | |
| Age (years) | <30 | 44 | 6.20 | (5.58; 7.63) | 44 | 4.16 | (3.41; 5.75) | 40 | 2.47 | (1.76; 3.59) |
|  | 30 - <40 | 61 | 6.95 | (6.00; 8.82) | 61 | 4.39 | (3.51; 5.03) | 57 | 2.43 | (1.72; 4.39) |
|  | 40 - <50 | 74 | 7.05 | (5.97; 8.89) | 74 | 4.01 | (3.30; 5.54) | 66 | 2.37 | (1.78; 4.13) |
|  | ≥ 50 | 59 | 8.19 | (6.21; 11.31) | 59 | 4.67 | (3.53; 6.19) | 54 | 2.23 | (1.65; 3.29) |
|  | | | | | | | | | | |
| Smoking status | Never | 54 | 6.64 | (6.08; 7.87) | 54 | 4.40 | (3.62; 5.08) | 48 | 2.26 | (1.65; 3.89) |
|  | Former | 62 | 7.29 | (5.50; 9.28) | 62 | 3.76 | (2.97; 5.08) | 58 | 2.70 | (1.72; 4.22) |
|  | Current | 122 | 7.11 | (6.06; 9.09) | 122 | 4.43 | (3.57; 5.70) | 111 | 2.39 | (1.78; 3.94) |
|  | | | | | | | | | | |
| Body mass index (kg/m²) | <30 | 171 | 7.01 | (5.97; 8.89) | 171 | 4.42 | (3.45; 5.56) | 155 | 2.48 | (1.72; 4.09) |
|  | ≥ 30 | 67 | 7.20 | (6.08; 9.28) | 67 | 3.89 | (3.30; 5.26) | 62 | 2.20 | (1.59; 3.23) |
|  | | | | | | | | | | |
| Serum ferritin (µg/L) | < 25 | 10 | 6.31 | (4.64; 8.33) | 10 | 4.29 | (2.93; 5.91) | 10 | 2.91 | (2.21; 3.29) |
|  | 25 - <300 | 185 | 6.64 | (5.72; 8.22) | 185 | 4.27 | (3.30; 5.15) | 167 | 2.29 | (1.72; 3.69) |
|  | 300 - <400 | 22 | 9.23 | (7.26; 11.18) | 22 | 4.54 | (3.33; 6.19) | 20 | 3.22 | (1.94; 4.49) |
|  | ≥ 400 | 21 | 10.88 | (9.03; 12.96) | 21 | 5.33 | (4.16; 6.30) | 20 | 2.40 | (1.78; 5.24) |
|  | | | | | | | | | | |
| Chromium in erythrocytes | Below limit of detection | 220 | 7.05 | (5.96; 9.04) | 220 | 4.33 | (3.31; 5.45) | 199 | 2.33 | (1.72; 3.55) |
|  | Above limit of detection | 15 | 6.86 | (6.08; 8.21) | 15 | 4.53 | (3.85; 5.33) | 15 | 3.65 | (1.54; 5.49) |
|  | | | | | | | | | | |
| C-reactive protein (mg/dL) | < 1 | 229 | 7.01 | (5.99; 8.97) | 229 | 4.28 | (3.39; 5.31) | 209 | 2.35 | (1.72; 3.78) |
|  | ≥ 1 | 9 | 8.40 | (6.10; 10.20) | 9 | 5.07 | (4.42; 6.29) | 8 | 3.14 | (1.66; 5.82) |
|  | | | | | | | | | | |
| Acetylsalicyl-acid containing | None | 228 | 7.00 | (5.98; 8.93) | 228 | 4.31 | (3.38; 5.35) | 208 | 2.35 | (1.72; 3.87) |
| medication | Yes | 10 | 8.38 | (6.13; 11.94) | 10 | 4.47 | (3.58; 4.97) | 9 | 2.97 | (1.73; 4.45) |
|  | | | | | | | | | | |
| Welding technique | Flux-cored arc welding | 45 | 6.86 | (5.97; 9.03) | 45 | 4.01 | (3.18; 4.79) | 43 | 4.58 | (3.23; 6.76) |
|  | Gas metal arc welding with massive wire | 96 | 7.22 | (5.73; 8.96) | 96 | 4.33 | (3.45; 5.58) | 95 | 2.28 | (1.65; 3.29) |
|  | Shielded metal arc welding | 19 | 7.72 | (5.51; 9.50) | 19 | 4.42 | (3.07; 5.37) | 19 | 2.86 | (1.38; 4.39) |
|  | Tungsten inert gas welding | 65 | 6.41 | (6.00; 8.25) | 65 | 4.41 | (3.45; 5.15) | 50 | 1.98 | (1.67; 2.43) |
|  | Miscellaneous | 13 | 10.06 | (7.74; 12.24) | 13 | 6.45 | (4.89; 7.63) | 10 | 1.68 | (0.88; 1.96) |
|  | | | | | | | | | | |
| Cr content of electrode or steel | ≤ 5 % | 91 | 7.34 | (6.08; 9.09) | 91 | 4.01 | (3.18; 5.08) | 91 | 3.22 | (2.17; 4.75) |
|  | > 5 % | 147 | 6.66 | (5.92; 8.89) | 147 | 4.42 | (3.53; 5.56) | 126 | 2.04 | (1.63; 3.13) |
|  | | | | | | | | | | |
| Physical workload | Low | 42 | 6.51 | (6.00; 8.40) | 42 | 4.67 | (3.62; 6.06) | 39 | 1.89 | (1.59; 2.35) |
|  | Medium | 149 | 7.20 | (5.96; 9.18) | 149 | 4.28 | (3.33; 5.22) | 131 | 2.33 | (1.65; 3.78) |
|  | High | 47 | 7.09 | (6.06; 9.09) | 47 | 4.13 | (3.51; 5.26) | 47 | 3.55 | (2.51; 5.49) |
|  | | | | | | | | | | |
| Season | Spring | 58 | 6.48 | (5.70; 8.77) | 58 | 3.95 | (3.30; 4.83) | 57 | 3.14 | (1.87; 5.01) |
|  | Summer | 96 | 6.73 | (5.73; 8.01) | 96 | 4.44 | (3.34; 5.24) | 96 | 2.26 | (1.69; 3.12) |
|  | Autumn | 66 | 8.29 | (6.54; 11.27) | 66 | 4.71 | (3.49; 6.19) | 54 | 3.00 | (1.89; 4.18) |
|  | Winter | 18 | 7.04 | (6.19; 9.50) | 18 | 4.06 | (3.08; 5.56) | 10 | 1.39 | (1.21; 1.73) |
|  | | | | | | | | | | |
