# Supplementary material for: Oxidatively damaged guanosine in white blood cells and in urine of welders: associations with exposure to welding fumes and body iron stores
Source: Arch Toxicol. 2014 Aug 9;89(8):1257–69. doi: 10.1007/s00204-014-1319-2 (PMC4508371; doi:10.1007/s00204-014-1319-2)
Supplement: Supplementary file 2 — Supplementary material 2 (DOCX 24 kb) [file 204_2014_1319_MOESM2_ESM.docx]

**Table S2: Correlations among exposure variables and adducts measures in welders**

|  |  | Kendall’s tau coefficient τ_b_ | P-value |
| --- | --- | --- | --- |
| 8-oxodGuo/10^6^dGuo, N=217 | 8-oxodGuo µg/g creatinine | -0.07 | 0.11 |
|  | 8-oxoGuo µg/g creatinine | 0.04 | 0.37 |
| 8-oxodGuo µg/g creatinine, N=238 | 8-oxoGuo µg/g creatinine | 0.40 | <.0001 |
| Welding fume (mg/m^3^), N=236 | Manganese (µg/m³) | 0.80 | <.0001 |
|  | Iron (µg/m³) | 0.76 | <.0001 |
|  | Chromium (µg/m^3^) | 0.12 | 0.009 |
|  | Nickel (µg/m³) | 0.24 | <.0001 |
| Iron (µg/m³), N=236 | Manganese (µg/m³) | 0.78 | <.0001 |
|  | Chromium (µg/m^3^) | 0.13 | 0.003 |
|  | Nickel (µg/m³) | 0.23 | <.0001 |
| Manganese (µg/m³), N=236 | Chromium (µg/m^3^) | 0.18 | <.0001 |
|  | Nickel (µg/m³) | 0.30 | <.0001 |
| Chromium (µg/m^3^), N=236 | Nickel (µg/m³) | 0.63 | <.0001 |
| Serum ferritin (µg/L), N=238 | Manganese in blood (µg/L) | 0.05 | 0.30 |
|  | Urinary chromium (µg/L) | -0.00 | 0.93 |
|  | Urinary nickel (µg/L) | 0.05 | 0.281 |
| Manganese in blood (µg/L), N=238 | Urinary chromium (µg/L) | 0.10 | 0.034 |
|  | Urinary nickel (µg/L) | 0.24 | <.0001 |
| Urinary chromium (µg/L), N=238 | Urinary nickel (µg/L) | 0.45 | <.0001 |
