# Supplementary material for: Oxidatively damaged guanosine in white blood cells and in urine of welders: associations with exposure to welding fumes and body iron stores
Source: Arch Toxicol. 2014 Aug 9;89(8):1257–69. doi: 10.1007/s00204-014-1319-2 (PMC4508371; doi:10.1007/s00204-014-1319-2)
Supplement: Supplementary file 3 — Supplementary material 3 (DOCX 20 kb) [file 204_2014_1319_MOESM3_ESM.docx]

**Table S3: Influence of respirable manganese and chromium and of other potential predictors on 8-oxodGuo and 8-oxoGuo in welders (random intercept models)**

|  |  | **Urinary 8-oxoGuo [µg/L]**  N=236 | | | **Urinary 8-oxodGuo [µg/L]**  N=236 | | | **8-oxodGuo/10^6^ dGuo**  N=215 | | |
| --- | --- | --- | --- | --- | --- | --- | --- | --- | --- | --- |
|  |  | *Exp* |  |  | *Exp* |  |  | *Exp* |  |  |
|  |  | *(coefficient)* | *95% CI* | *P-value* | *(coefficient)* | *95% CI* | *P-value* | *(coefficient)* | *95% CI* | *P-value* |
| **Fixed Effects** |  |  |  |  |  |  |  |  |  |  |
| Intercept |  | 1.83 | (0.96 – 3.48) | 0.063 | 2.41 | (1.21 – 4.79) | 0.015 | 2.85 | (1.34 – 6.07) | 0.0087 |
| Manganese | < LOQ (N=5) | 1.12 | (0.82 – 1.53) | 0.47 | 0.97 | (0.70 – 1.35) | 0.86 | 0.82 | (0.59 – 1.14) | 0.23 |
| [µg/m³] | ≥ LOQ & ≤ 9.7 µg/m³ (N=57/54) | 1 |  |  | 1 |  |  | 1 |  |  |
|  | 9.7 – 66 µg/m³ (N=59/47) | 1.06 | (0.92 – 1.22) | 0.41 | 0.98 | (0.84 – 1.14) | 0.76 | 1.01 | (0.85 – 1-20) | 0.93 |
|  | 66 – 320 µg/m³ (N=61/56) | 1.16 | (1.00 – 1.34) | 0.049 | 1.05 | (0.90 – 1.23) | 0.54 | 0.94 | (0.77 – 1.14) | 0.52 |
|  | > 320 µg/m³ (N=54/53) | 1.19 | (1.02 – 1.38) | 0.026 | 0.97 | (0.82 – 1.15) | 0.71 | 0.99 | (0.80 – 1.23) | 0.92 |
| Chromium | < LOQ (N=55/55) | 0.84 | (0.74 – 0.96) | 0.0075 | 0.85 | (0.74 – 0.98) | 0.022 | 0.84 | (0.72 – 0.98) | 0.030 |
| [µg/m^3^] | ≥ LOQ & ≤ 6.8 µg/m³ (N=91/83) | 1 |  |  | 1 |  |  | 1 |  |  |
|  | > 6.8 µg/m³ (N=90/77) | 0.96 | (0.85 – 1.08) | 0.48 | 0.97 | (0.86 – 1.10) | 0.66 | 1.04 | (0.89 – 1.21) | 0.63 |
| Ln urinary creatinine (g/L) | | 2.54 | (2.37 – 2.71) | <.0001 | 2.48 | (2.31 – 2.66) | <.0001 |  |  |  |
| Active smokers (N=121/110) *vs*. non-smokers (N=115/105) | | 1.08 | (0.98 – 1.18) | 0.12 | 1.14 | (1.04 – 1.26) | 0.0070 | 0.96 | (0.88 – 1.06) | 0.44 |
| Ln age [years] | | 1.44 | (1.23 – 1.70) | <.0001 | 1.18 | (0.99 – 1.40) | 0.066 | 0.99 | (0.83 – 1.19) | 0.90 |
|  |  |  |  |  |  |  |  |  |  |  |
| **Random Effects** | | *Variance component* | *95% CI* | *P-value* | *Variance component* | *95% CI* | *P-value* | *Variance component* | *95% CI* | *P-value* |
| Level-two variance estimate (between plants) | | 0.010 | (0.005 – 0.068) | 0.065 | 0.018 | (0.008 – 0.077) | 0.032 | 0.24 | (0.14 – 0.54) | 0.0018 |
| Level-one variance estimate (within plants) | | 0.103 | (0.086 – 0.127) | <.0001 | 0.115 | (0.095 – 0.141) | <.0001 | 0.11 | (0.09 – 0.13) | <.0001 |
|  |  |  |  |  |  |  |  |  |  |  |
