# Supplementary material for: Oxidatively damaged guanosine in white blood cells and in urine of welders: associations with exposure to welding fumes and body iron stores
Source: Arch Toxicol. 2014 Aug 9;89(8):1257–69. doi: 10.1007/s00204-014-1319-2 (PMC4508371; doi:10.1007/s00204-014-1319-2)
Supplement: Supplementary file 4 — Supplementary material 4 (DOCX 20 kb) [file 204_2014_1319_MOESM4_ESM.docx]

**Table S4: Influence of manganese in blood, urinary chromium and other potential predictors on urinary 8-oxodGuo and 8-oxoGuo in welders (random intercept models)**

|  | |  | **Urinary 8-oxoGuo (µg/L)**  N=238 | | | **Urinary 8-oxodGuo (µg/L)**  N=238 | | | **8-oxodGuo/10^6^ dGuo**  N=217 | | |
| --- | --- | --- | --- | --- | --- | --- | --- | --- | --- | --- | --- |
|  | |  | *Exp* |  |  | *Exp* |  |  | *Exp* |  |  |
|  | |  | *(coefficient)* | *95% CI* | *P-value* | *(coefficient)* | *95% CI* | *P-value* | *(coefficient)* | *95% CI* | *P-value* |
| **Fixed Effects** | |  |  |  |  |  |  |  |  |  |  |
| Intercept | |  | 1.59 | (0.79 – 3.21) | 0.19 | 2.19 | (1.03 – 4.67) | 0.043 | 2.46 | (1.05 – 5.76) | 0.038 |
| Ln manganese in blood (µg/L) | |  | 1.17 | (1.04 – 1.31) | 0.011 | 1.07 | (0.94 – 1.21) | 0.33 | 1.04 | (0.90 – 1.20) | 0.59 |
| Urinary chromium (µg/L) | | < LOQ (N=106/103) | 0.90 | (0.79 – 1.03) | 0.12 | 0.97 | (0.84 – 1.12) | 0.70 | 1.07 | (0.91 – 1.25) | 0.43 |
|  | | ≥ LOQ & ≤ 1.695 µg/L (N=33/27) | 1 |  |  | 1 |  |  | 1 |  |  |
|  | | 1.695 - 2.825 µg/L (N=33/28) | 0.98 | (0.84 – 1.16) | 0.84 | 1.08 | (0.91 – 1.28) | 0.40 | 1.08 | (0.90 – 1.30) | 0.42 |
|  | | 2.825 - 7.850 µg/L (N=33/31) | 1.17 | (0.99 – 1.39) | 0.068 | 1.20 | (1.00 – 1.43) | 0.055 | 1.13 | (0.91 – 1.39) | 0.26 |
|  | | > 7.850 µg/L (N=33/28) | 1.06 | (0.89 – 1.26) | 0.49 | 1.10 | (0.91 – 1.33) | 0.31 | 1.14 | (0.92 – 1.42) | 0.24 |
| Ln urinary creatinine (g/L) | | | 2.48 | (2.32 – 2.66) | <.0001 | 2.41 | (2.24 – 2.60) | <.0001 |  |  |  |
| Current smokers (N=122/111) vs. non-smokers (N=116/106) | | | 1.08 | (0.99 – 1.18) | 0.080 | 1.14 | (1.04 – 1.26) | 0.0048 | 0.97 | (0.88 – 1.07) | 0.56 |
| Ln age [years] | | | 1.38 | (1.18 – 1.62) | <.0001 | 1.13 | (0.96 – 1.35) | 0.15 | 0.97 | (0.81 – 1.16) | 0.73 |
|  |  | |  |  |  |  |  |  |  |  |  |
| **Random Effects** | | | *Variance component* | *95% CI* | *P-value* | *Variance component* | *95% CI* | *P-value* | *Variance component* | *95% CI* | *P-value* |
| Level-two variance estimate (between plants) | | | 0.011 | (0.004 – 0.058) | 0.048 | 0.014 | (0.006 - 0.069) | 0.041 | 0.23 | (0.13 – 0.50) | 0.0011 |
| Level-one variance estimate (within plants) | | | 0.100 | (0.083 – 0.122) | <.0001 | 0.115 | (0.095 - 0.140) | <.0001 | 0.11 | (0.09 – 0.14) | <.0001 |
|  |  | |  |  |  |  |  |  |  |  |  |
