# Supplementary material for: Oxidatively damaged guanosine in white blood cells and in urine of welders: associations with exposure to welding fumes and body iron stores
Source: Arch Toxicol. 2014 Aug 9;89(8):1257–69. doi: 10.1007/s00204-014-1319-2 (PMC4508371; doi:10.1007/s00204-014-1319-2)
Supplement: Supplementary file 5 — Supplementary material 5 (DOCX 20 kb) [file 204_2014_1319_MOESM5_ESM.docx]

**Table S5: Influence of respirable manganese and nickel and of other potential predictors on 8-oxodGuo and 8-oxoGuo in welders (random intercept models)**

|  |  | **Urinary 8-oxoGuo [µg/L]**  N=236 | | | **Urinary 8-oxodGuo [µg/L]**  N=236 | | | **8-oxodGuo/10^6^ dGuo**  N=215 | | |
| --- | --- | --- | --- | --- | --- | --- | --- | --- | --- | --- |
|  |  | *Exp* |  |  | *Exp* |  |  | *Exp* |  |  |
|  |  | *(coefficient)* | *95% CI* | *P-value* | *(coefficient)* | *95% CI* | *P-value* | *(coefficient)* | *95% CI* | *P-value* |
| **Fixed Effects** |  |  |  |  |  |  |  |  |  |  |
| Intercept |  | 1.81 | (0.94 – 3.48) | 0.073 | 2.39 | (1.19 – 4.78) | 0.016 | 2.76 | (1.29 – 5.91) | 0.011 |
| Manganese | < LOQ (N=5) | 1.08 | (0.79 – 1.48) | 0.62 | 0.94 | (0.68 – 1.31) | 0.73 | 0.80 | (0.57 – 1.10) | 0.17 |
| [µg/m³] | ≥ LOQ & ≤ 9.7 µg/m³ (N=57/54) | 1 |  |  | 1 |  |  | 1 |  |  |
|  | 9.7 – 66 µg/m³ (N=59/47) | 1.06 | (0.93 – 1.21) | 0.39 | 0.98 | (0.85 – 1.13) | 0.79 | 1.03 | (0.88 – 1.21) | 0.71 |
|  | 66 – 320 µg/m³ (N=61/56) | 1.14 | (0.98 – 1.32) | 0.088 | 1.04 | (0.89 – 1.22) | 0.64 | 0.95 | (0.79 – 1.15) | 0.60 |
|  | > 320 µg/m³ (N=54/53) | 1.20 | (1.03 – 1.40) | 0.020 | 0.98 | (0.83 – 1.16) | 0.81 | 1.04 | (0.84 – 1.28) | 0.73 |
| Nickel | < LOQ (N=76/75) | 0.95 | (0.84 – 1.07) | 0.39 | 0.93 | (0.81 – 1.06) | 0.27 | 0.94 | (0.80 – 1.09) | 0.39 |
| [µg/m^3^] | ≥ LOQ & ≤ 8.4 µg/m³ (N=81/67) | 1 |  |  | 1 |  |  | 1 |  |  |
|  | > 8.4 µg/m³ (N=79/73) | 1.01 | (0.89 – 1.14) | 0.92 | 1.00 | (0.88 – 1.14) | 0.99 | 1.05 | (0.91 – 1.22) | 0.49 |
| Ln urinary creatinine (g/L) | | 2.55 | (2.39 – 2.73) | <.0001 | 2.50 | (2.33 – 2.68) | <.0001 |  |  |  |
| Active smokers (N=121/110) *vs*. non-smokers (N=115/105) | | 1.08 | (0.99 – 1.18) | 0.099 | 1.15 | (1.04 – 1.26) | 0.0060 | 0.97 | (0.88 – 1.07) | 0.57 |
| Ln age [years] | | 1.43 | (1.21 – 1.69) | <.0001 | 1.17 | (0.98 – 1.39) | 0.079 | 0.98 | (0.82 – 1.18) | 0.86 |
|  |  |  |  |  |  |  |  |  |  |  |
| **Random Effects** | | *Variance component* | *95% CI* | *P-value* | *Variance component* | *95% CI* | *P-value* | *Variance component* | *95% CI* | *P-value* |
| Level-two variance estimate (between plants) | | 0.009 | (0.003 – 0.073) | 0.075 | 0.018 | (0.008 – 0.079) | 0.035 | 0.23 | (0.13 – 0.51) | 0.0017 |
| Level-one variance estimate (within plants) | | 0.107 | (0.089 – 0.131) | <.0001 | 0.117 | (0.097 – 0.143) | <.0001 | 0.11 | (0.09 – 0.14) | <.0001 |
|  |  |  |  |  |  |  |  |  |  |  |
