# Supplementary material for: Oxidatively damaged guanosine in white blood cells and in urine of welders: associations with exposure to welding fumes and body iron stores
Source: Arch Toxicol. 2014 Aug 9;89(8):1257–69. doi: 10.1007/s00204-014-1319-2 (PMC4508371; doi:10.1007/s00204-014-1319-2)
Supplement: Supplementary file 6 — Supplementary material 6 (DOCX 20 kb) [file 204_2014_1319_MOESM6_ESM.docx]

**Table S6: Influence of manganese in blood, urinary nickel and other potential predictors on urinary 8-oxodGuo and 8-oxoGuo in welders (random intercept models)**

|  | |  | **Urinary 8-oxoGuo (µg/L)**  N=238 | | | **Urinary 8-oxodGuo (µg/L)**  N=238 | | | **8-oxodGuo/10^6^ dGuo**  N=217 | | |
| --- | --- | --- | --- | --- | --- | --- | --- | --- | --- | --- | --- |
|  | |  | *Exp* |  |  | *Exp* |  |  | *Exp* |  |  |
|  | |  | *(coefficient)* | *95% CI* | *P-value* | *(coefficient)* | *95% CI* | *P-value* | *(coefficient)* | *95% CI* | *P-value* |
| **Fixed Effects** | |  |  |  |  |  |  |  |  |  |  |
| Intercept | |  | 1.44 | (0.70 – 2.96) | 0.31 | 2.05 | (0.95 – 4.41) | 0.065 | 2.44 | (1.05 – 5.64) | 0.039 |
| Ln manganese in blood (µg/L) | |  | 1.14 | (1.01 – 1.28) | 0.040 | 1.05 | (0.93 – 1.20) | 0.43 | 1.07 | (0.93 – 1.23) | 0.36 |
| Urinary nickel (µg/L) | | < LOQ (N=73/68) | 0.96 | (0.84 – 1.10) | 0.60 | 1.03 | (0.90 – 1.19) | 0.65 | 0.93 | (0.80 – 1.09) | 0.38 |
|  | | ≥ LOQ & ≤ 2.63 µg/L (N=42/40) | 1 |  |  | 1 |  |  | 1 |  |  |
|  | | 2.63 – 4.12 µg/L (N=41/38) | 1.03 | (0.89 – 1.20) | 0.71 | 0.95 | (0.81 – 1.11) | 0.48 | 1.12 | (0.95 – 1.32) | 0.16 |
|  | | 4.12 – 8.04 µg/L (N=41/37) | 1.10 | (0.95 – 1.28) | 0.22 | 1.06 | (0.90 – 1.25) | 0.47 | 0.96 | (0.81 – 1.13) | 0.63 |
|  | | > 8.04 µg/L (N=41/34) | 1.16 | (0.98 – 1.38) | 0.086 | 1.19 | (0.99 – 1.44) | 0.070 | 0.94 | (0.76 – 1.17) | 0.57 |
| Ln urinary creatinine (g/L) | | | 2.49 | (2.32 – 2.68) | <.0001 | 2.45 | (2.28 – 2.65) | <.0001 |  |  |  |
| Current smokers (N=122/111) vs. non-smokers (N=116/106) | | | 1.08 | (0.99 – 1.18) | 0.10 | 1.15 | (1.05 – 1.26) | 0.0032 | 0.96 | (0.87 – 1.05) | 0.37 |
| Ln age [years] | | | 1.42 | (1.21 – 1.67) | <.0001 | 1.16 | (0.98 – 1.37) | 0.091 | 0.98 | (0.82 – 1.18) | 0.86 |
|  |  | |  |  |  |  |  |  |  |  |  |
| **Random Effects** | | | *Variance component* | *95% CI* | *P-value* | *Variance component* | *95% CI* | *P-value* | *Variance component* | *95% CI* | *P-value* |
| Level-two variance estimate (between plants) | | | 0.010 | (0.004 – 0.063) | 0.062 | 0.019 | (0.009 - 0.066) | 0.018 | 0.22 | (0.13 – 0.47) | 0.0011 |
| Level-one variance estimate (within plants) | | | 0.103 | (0.086 – 0.127) | <.0001 | 0.112 | (0.093 - 0.137) | <.0001 | 0.11 | (0.09 – 0.13) | <.0001 |
|  |  | |  |  |  |  |  |  |  |  |  |
